# Current state of molecular and metabolic strategies for the improvement of L-asparaginase expression in heterologous systems

**DOI:** 10.3389/fphar.2023.1208277

**Published:** 2023-06-22

**Authors:** Nicolás Lefin, Javiera Miranda, Jorge F. Beltrán, Lisandra Herrera Belén, Brian Effer, Adalberto Pessoa, Jorge G. Farias, Mauricio Zamorano

**Affiliations:** ^1^ Department of Chemical Engineering, Science and Engineering Faculty, Universidad de La Frontera, Temuco, Chile; ^2^ Departamento de Ciencias Básicas, Facultad de Ciencias, Universidad Santo Tomas, Santiago, Chile; ^3^ Center of Excellence in Translational Medicine and Scientific and Technological Bioresource Nucleus, Universidad de La Frontera, Temuco, Chile; ^4^ Department of Biochemical and Pharmaceutical Technology, School of Pharmaceutical Sciences, University of São Paulo, São Paulo, Brazil

**Keywords:** L-asparaginase, molecular strategies, rational design, heterologous expression system, industrial bioprocessing

## Abstract

Heterologous expression of L-asparaginase (L-ASNase) has become an important area of research due to its clinical and food industry applications. This review provides a comprehensive overview of the molecular and metabolic strategies that can be used to optimize the expression of L-ASNase in heterologous systems. This article describes various approaches that have been employed to increase enzyme production, including the use of molecular tools, strain engineering, and *in silico* optimization. The review article highlights the critical role that rational design plays in achieving successful heterologous expression and underscores the challenges of large-scale production of L-ASNase, such as inadequate protein folding and the metabolic burden on host cells. Improved gene expression is shown to be achievable through the optimization of codon usage, synthetic promoters, transcription and translation regulation, and host strain improvement, among others. Additionally, this review provides a deep understanding of the enzymatic properties of L-ASNase and how this knowledge has been employed to enhance its properties and production. Finally, future trends in L-ASNase production, including the integration of CRISPR and machine learning tools are discussed. This work serves as a valuable resource for researchers looking to design effective heterologous expression systems for L-ASNase production as well as for enzymes production in general.

## 1 Introduction

**FIGURE 1:**
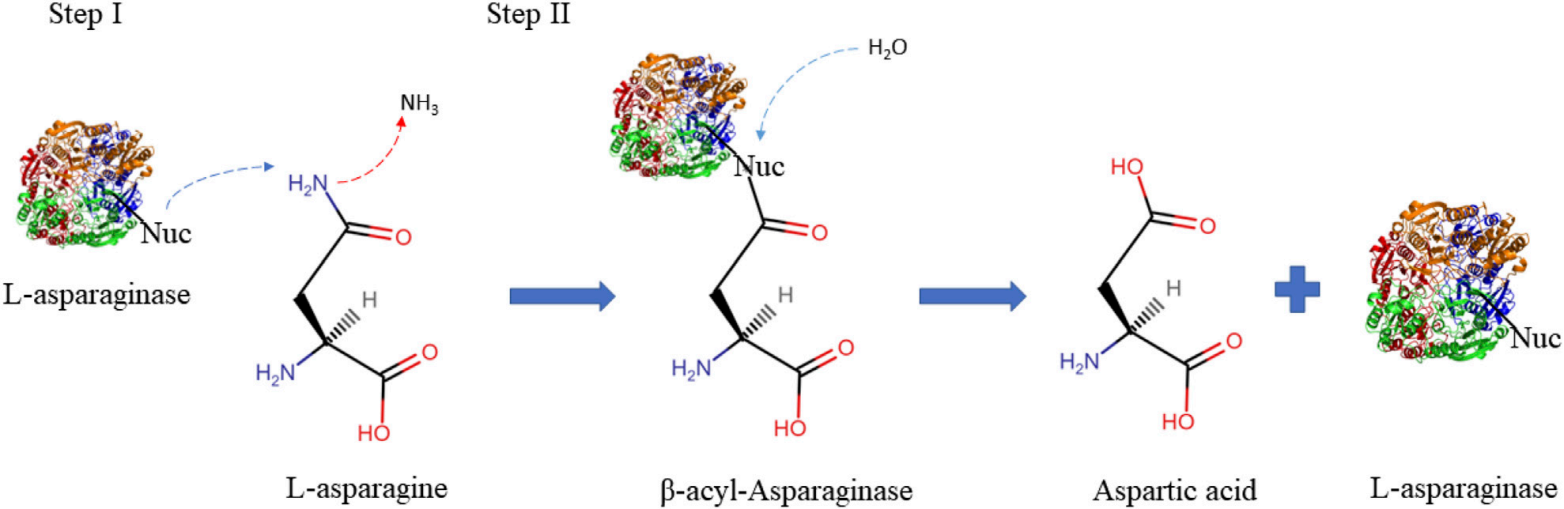
Schematic illustration describing the two steps L-asparaginase reaction mechanisms. The first step consists of activating the enzyme’s nucleophilic residue with the strong base NH2, which then attacks the l-asparagine amide carbon atom to produce the beta-acyl-enzyme intermediate. The second step involves activating the nucleophile with a water molecule, which attacks the ester carbon repeatedly to produce l-aspartic acid and release ammonia (Nunes et al., 2020). Nuc: nucleophilic residues.

L-asparaginase amidohydrolase (L-ASNase), also known as aminohydrolase pertains to the amidase group of enzymes (EC3.5.1.1), is widely recognized as one of the main anticancer drugs and a promising acrylamide mitigator in the food industry. This is due to its role in the hydrolysis of L-asparagine by a two-step mechanism where first the nucleophilic residue (Nuc) attacks the amide carbon atom of L-asparagine, releasing ammonia, thus generating a beta-acyl-enzyme intermediate. Subsequently, it acts on the ester carbon mediated by a water molecule, forming the L-aspartate molecule as shown in Figure 1 (Shakambari et al., 2019; Chand et al., 2020).

The foundation of ASNase-based treatments is the starvation of amino acids principle (Batool et al., 2016). Due to a mutation in the gene encoding L-asparagine synthetase, many leukemia or lymphoma cells cannot synthesize L-asparaginase and rely on its supply from plasma (Broome, 1968). As a result, leukemia cells undergo starvation and subsequent apoptosis due to altered signaling pathways caused by decreased plasma L-asparagine levels, which are hydrolyzed by L-ASNase (Ueno et al., 1997). This makes L-ASNase a key chemotherapeutic agent for the treatment of acute lymphoblastic leukemia (ALL), and lymphosarcoma. Additionally, its use has been reported in the treatment of acute myelomonocytic leukemia, critical lymphoblastic leukemia, melogenic leukemia, Hodgkin’s lymphoma, chronic lymphocytic leukemia, and more (Vimal et al., 2018). Moreover, L-ASNase has several non-medical applications, particularly as a mitigation agent for acrylamide, which is a known carcinogen (level 2 A) and an important neurotoxin (Wang et al., 2021). Acrylamide forms between reducing sugars, and amino acids (such as L-asparagine) when starchy foods are cooked at temperatures over 120°C, and under low humidity. This non-enzymatic mechanism is known as the Maillard reaction. The process involves the formation of a Schiff base and its subsequent decarboxylation. When this progression occurs under heat, either an ammonia or an imine molecule is eliminated, and subsequently replaced to form acrylamide (Jia et al., 2021). Consequently, when L-ASNase is administered, it hydrolyses the present L-asparagine and forms aspartic acid. As a result, the Maillard reaction cannot progress, and the formation of acrylamide is inhibited (Kornbrust et al., 2009). In 2016, the European Food and Drink Federation published a strategy called *“Toolbox Acrylamide”*, driven by industrial enzymes, which promotes the reduction of residual acrylamide in foods to protect public health.

L-ASNase is one of the therapeutic enzymes with the highest global production. It contributes 40% of the total global demand for enzymes with general use. In addition, it represents approximately one-third of the world’s requirement for antileukemic and antilymphoma agents. Thus it is one of the enzymatic products with major industrial potential (Vimal and Kumar, 2017). ln 2017, its global demand was approximately USD 380 m, and it is estimated to reach USD 420 m by 2025 (Alam et al., 2019). Currently, there are various formulations of L-asparaginase available in the market for clinical use, including those of bacterial origin such as native, PEGylated, and recombinant L-asparaginases from *Escherichia coli* (*E. coli*), as well as native L-asparaginases from *Erwinia chrysanthemi* (*E. chrysanthemi*). Additionally, fungal-derived asparaginases approved for food use, such as those from *Aspergillus oryzae* and *Aspergillus niger*, are also available (Battistel et al., 2021; Jia et al., 2021). All these formulations have been tested to improve their safety profiles (Burke, 2014; Effer et al., 2020; de Almeida Parizotto et al., 2021). However, Due to standard l-asparaginase preparations carries low thermostability, occurrence of side effects and restricted substrate specificity, these applications have been hampered by the diverse conditions frequently seen in the food and healthcare sectors (Zhang et al., 2021). Hence, it is of paramount importance to search for products that allow for the improvement of their properties, such as increased l-ASNase activity, reduced glutaminase activity, and stability for human physiological conditions in the therapeutic case, and improved L-ASNase activity and thermal stability for the food industry, while carefully considering the composition employed in their production, such as the immunological effects triggered by the bacterial-derived L-ASNase itself (Kishore et al., 2015; Wang et al., 2021).

Currently, several reviews have been attempted compiling L-Asparaginases from various sources that are able to improve both pharmacokinetics, reduce side effects and stability (Batool et al., 2016; Wang et al., 2021; Patel et al., 2022). Additionally, thanks to the principles of Quality by Design (QbD), several techniques have been proposed to overcome the disadvantages of the treatment, allowing the development of L-ASNases “bio-betters” (Brumano et al., 2019; Nunes et al., 2020). Each of these attempts aims to express these L-ASNases in heterologous hosts that allow them to be produced efficiently, economically, and easily. However, expressing a different or modified host protein from a different organism presents several challenges.

**FIGURE 2:**
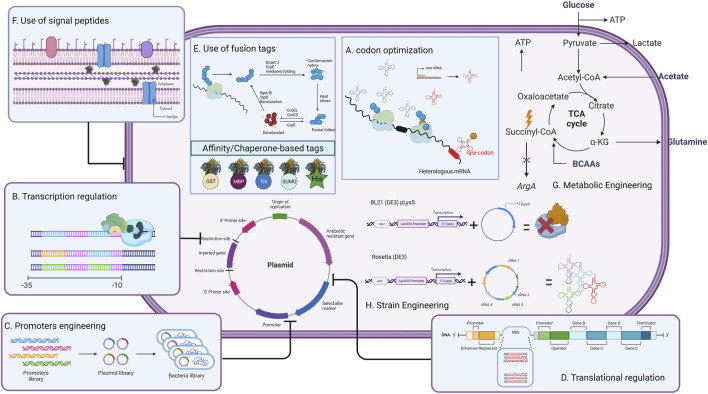
General strategies to improve L-ASNase production in heterologous host systems. This strategy can be modified depending on the difficulties that may arise during expression.

In this review, the main genetic modifications and current strategies developed to improve L-ASNase expression in microbial heterologous systems, including codon optimization, transcriptional regulation, promoter engineering, translation regulation, optimization of factors affecting expression, and host strains genetic and metabolic engineering (Figure 2), will be discussed. Special attention will be paid to *E. coli*, *Bacillus subtilis* and *P. pastoris*, which are the preferred hosts for L-ASNase expression. Finally, future challenges for the rational design of heterologous systems for L-ASNase expression are discussed.

## 2 Type and characteristics of L-ASNase in organisms

**FIGURE 3:**
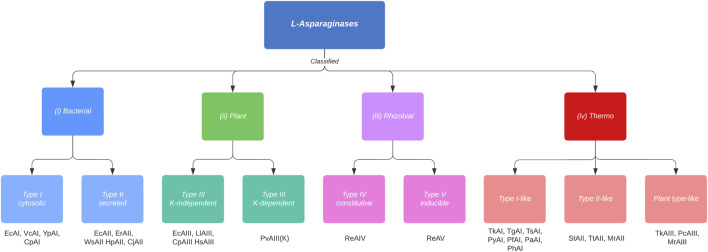
Classification and types of L-asparaginases based on the new criteria proposed by Loch and Jaskolski (Loch and Jaskolski, 2021), adding the thermophiles classification (Class IV) proposed by Dumina and Zhgun (Dumina and Zhgun, 2023). Cytosolic Class I, type I enzymes are constitutively expressed, whereas the expression of type II enzymes, which are secreted into the periplasm, is induced under anaerobic conditions. Class II enzymes have dimeric structure, which are subdivided into potassium-dependent and potassium-independent type III enzymes. This dependence arises due to a K^+^ coordination to the side chain of Arginine 104, which allows anchoring of the substrate to the active site. In Rizhobial Class III, constitutive type IV enzymes are thermostable, whereas type V enzymes are considered thermolabile and their expression is induced by the presence of l-Asn. Class IV (thermophiles) enzymes are subclassified into type I-like, type II-like and type plant-like. Type I-like enzymes differ from mesophiles in their structure (thermophiles are homodimers) and in their amino acid identity (<37% identity). Type II-like enzymes reveal a rather reduced identity compared to mesophylls and they form structures different from mesophylls (homodimeric and hexameric structures). Finally, the plant-like types present dimeric structures, like mesophylls, however, they can withstand high temperatures due to their amino acid divergence (<50% identity). Examples of enzymes are listed below the boxes. The organism name abbreviations are as follows: Ec, Esherichia coli; Pf, Pyrococcus furiosus; Ph, Pyrococcus horikoshii; Py, Pyrococcus yayanossi; Pa, Pyrococcus abyssi; Pc, Pyrobaculum calidifontis; St, *Streptomyces* thermoletus; Tt, Thermus thermophilus; Tk, Thermococcus kodakarensis; Tz, Thermococcus zilligii; Tg, Thermococcus gammatolerans; Ts, Thermococcus sibiricus; Mr, Melioribacter roseus; Vc, *Vibrio cholerae*; Yp, *Yersinia pestis*; Cp, *Cavia porcellus*; Er, *Erwinia* chrysanthemi; Ew, *Erwinia* carotovora; Cj, *Campylobacter* jejuni; Hp, *Helicobacter pylori*; Ws, Wolinella succinogenes; Ll, Lupinus luteus; Pv, Phaseolus vulgaris; Hs, *Homo sapiens*; Re, Rhizobium etli.

L-ASNase is widespread, and can be found in animals, plants, and microorganisms such as bacteria, fungi, and yeasts, and even in various thermophilic organisms. Any of these organisms can be a potential source for L-ASNase production. However, not all sources are equivalent (Castro et al., 2021). L-ASNase has been conventionally classified according to amino acid sequence, the organism expressing it, inducibility, cellular localization, substrate affinity, and quaternary structure into three families: 1) bacterial, 2) plant, and 3) rhizobial (Michalska and Jaskolski, 2006; Loch and Jaskolski, 2021). However, this classification has been disputed due to the absence of the thermophilic group. According to Dumina et al. (Dumina et al., 2021), thermophilic L-ASNases differ from mesophiles both in their structural properties (they can be found in a hexameric form (Pritsa and Kyriakidis, 2001)), topological properties, deviation in the canonical arrangement of their active site, etc. For this reason, a new classification for thermophilic L-ASNases (class IV) proposed by the authors would be included in this review (Figure 3). Bacterial class L-ASNases can be subdivided into two different isozymes: Type I and II L-ASNases. Type I L-ASNases (EcA I) is a homodimeric constitutive enzyme located in the cytoplasm, with low affinity for L-asparagine and high affinity for L-glutamine (Pokrovskaya et al., 2022). Among the Type I enzymes, L-ASNases produced by *B. subtilis* (Jia et al., 2013; Feng et al., 2017; Jia et al., 2021; Niu et al., 2021), *Thermococcus kodakarensis* (Chohan and Rashid, 2013; Hong et al., 2014), and *Acinetobacter soli* (Jiao et al., 2020) are the most studied examples. Type II bacterial L-ASNases (EcAII), normally a homotetrameric form and located in periplasmic space with expression induced during anaerobiosis and are secreted only when bacteria are exposed to low nitrogen concentrations (Verma et al., 2007). Their properties have been discussed in *E. coli, Erwinia carotova, Erwinia chrysantemi, Saccharomyces cerevisiae,* etc (Yano et al., 2008). Although both isozymes exhibit enzymatic activity for l-asparagine and l-glutamine, their affinity for L-asparagine is what distinguishes them from one another. Since EcAII has a higher specific affinity for l-asparagine, which results in high antitumor activity and is therefore the one used in medicinal applications (Sharafi et al., 2017). To get an idea, the EcAII *K*_*M*_ (Michaelis-Menten constant) = 10–15 µM *versus* EcAI *K*_*M*_ = 3.5 mM. This means that enzymes EcAII display much higher (2+ orders of magnitude) affinity for L-asparaginase than EcAI (Nunes et al., 2020). Microbial enzymes, like EcAII, are more suitable than their animal and plant counterparts as they provide a consistent profile, stability, relative ease of production and purification, High yields and consistency; simplifying the modification and optimization of the manufacturing process (Lopes et al., 2017; Vachher et al., 2021).

Commercially, there are two products currently available for acrylamide mitigation in the food industry. These are PreventASe TM from DSM (Heerlen, Netherlands) and Acrylaway^®^ from Novozymes A/S (Bagsvaerd, Denmark). PreventASe TM was the first, launched in 2007. It was obtained after analyzing the gene sequence of *Aspergillus niger*. It has an acidic profile (optimum pH 4-5, temperature 50°C). Acrylaway^®^, on the other hand, is obtained from *Aspergillus oryzae* and has a near-neutral profile (pH optimum 7, temperature 37°C) (Xu et al., 2016). As for its safety, it has been observed over the years that there are no hazards in its use and these are recognized as safe by the U.S. government and are currently used in several countries, including the U.S., Australia, China, Russia, Mexico, etc. (Xu et al., 2016). Additionally, several products are currently available for anti-leukemia treatment. Elspar^®^, Oncaspar^®^ (Pegaspargase), Crisantaspase^®^, Kidrolase^®^, and Erwinase^®^ or Erwinaze^®^ are some of the commercially available brands of ASNase. Elspar^®^ contains L-asparaginase derived from *E. coli*. Oncaspar^®^ is a modified version of Elspar^®^ obtained by covalent conjugation of *E. coli* asparaginase with monomethoxypolyethyleneglycol (PEG), to increase the plasma half-life and decrease the immunogenicity and antigenicity of L-asparaginase. However, a higher prevalence of side effects has been observed (Galindo-Rodríguez et al., 2017). Crisantaspase^®^ and Erwinase^®^ are obtained from *Erwinia chrysanthemi*. The former is often used in combination with other anticancer drugs, while the latter is used in conjunction with chemotherapy or radiotherapy as part of treatment protocols. *Escherichia coli* Kidrolase^®^ is used in the treatment of ALL, leukemic meningitis, and non-Hodgkin’s lymphoma (Izadpanah Qeshmi et al., 2018). All these formulations have been tested to improve their safety (Burke, 2014; Effer et al., 2020; de Almeida Parizotto et al., 2021; Munhoz Costa et al., 2022).

Nevertheless, microbial L-ASNase II presents several problems when administered as an antileukemic drug, including severe allergic reactions, nausea, diabetes, pancreatitis, and venous thromboembolism (Goyal and Bhatt, 2015; Schmiegelow et al., 2016).

## 3 The search for the production of an improved heterologous L-ASNase with commercial value

In recent years, several biological sources of L-ASNase able to tackle some of the mentioned issues through engineering have been explored. These endeavors have yielded L-ASNases with. 1) reduced immunogenic activity or allergic reactions, 2) high catalytic activity, and 3) low-cost up and downstream processing (Patel et al., 2022). It has been observed that L-ASNases in eukaryotes, such as fungi and yeasts, can result in enzymes with fewer adverse effects and advantageous characteristics (Cachumba et al., 2016). For example, L-ASNase I from the yeast *Saccharomyces cerevisiae* (ScASNaseI) and expressed in *E. coli* BL21 (DE3) has been studied (Munhoz Costa et al., 2016). This is because it was predicted to be a bacterial type II isoform, being a possible candidate as an antileukemic agent. The specific activity of L-asparagine in ScASNaseI was 196.2 U/mg, a value similar to the commercial ASNase activity of *E. coli* (223 U/mg). This enzyme maintains allosteric behavior and localization in the cytosol of the enzyme, as in the case of type I enzymes, but with a *k*_*M*_ of 75 µM as in type II enzymes. In addition, they performed specific activity tests for L-glutamine, presenting 0.38% of L-asparaginase activity, and cytotoxicity tests on MOLT-4 leukemia cells, killing 85% of the cells under physiological conditions (pH 7.4 at 37°C), with optimal activity at pH 8.6 and at 40°C. This enzyme is compatible with the treatment of leukemia. However, alternative sources to bacteria and fungi are currently being explored (Patel et al., 2022). For example, marine microorganisms would produce L-ASNases capable of withstanding pH, salinity, and pressure conditions similar to those of blood plasma. (Qeshmi et al., 2018). A study focused on the cloning, expression, and characterization of L-asparaginase from marine *Pseudomonas aeruginosa* HR03 isolated from fish intestines in *E. coli* BL21 (D3) as a host. The recombinant L-asparaginase (HR03Asnase) was purified and its enzymatic properties were determined. The maximum activity of the enzyme was observed at 40°C and pH 8. The study suggests that HR03Asnase has potential for commercial applications in the food and health industries (Izadpanah Qeshmi et al., 2022). Additionally, thermophilic microorganisms would have been studied which can produce L-ASNases that remain stable at high temperatures, which are potentially suitable for the food industry (Dumina and Zhgun, 2023). A study, identifies a new thermostable L-asparaginase from *Pyrococcus yayanosii* CH1 expressed in *B. subtilis* 168. This L-ASNase was characterized by obtaining a maximum volumetric yield of 1483.81 U/mg, a maximum activity at 95°C and pH 8, making it suitable for industrial food application (Li et al., 2018).

In addition to this, several techniques have been proposed to overcome the disadvantages of native ASNases, improving it obtaining novel bio-betters ASNases. The term bio-better, refers to creating novel drugs by enhancing the features of current peptide- or protein-based biopharmaceuticals, such as affinity, selectivity, immunogenicity, and stability against degradation of proteases (Beck, 2011; Lagassé et al., 2017). These proteins are manufactured from molecular and/or chemical modifications of an original product to improve drug characteristics (Courtois et al., 2015). Molecular strategies like protein engineering by bioinformatics analysis, docking, molecular dynamics and site-directed mutagenesis have been mentioned among the most sophisticated techniques (Nguyen et al., 2016a; Ardalan et al., 2018; Mundaganur et al., 2014; Munhoz Costa et al., 2022). An investigation, by means of directed evolution methodology, succeeded in obtaining a double mutant ASNase from *E. chrysanthemi* expressed in *E. coli* BL21 (DE3). This mutant L-ASNase, besides having a specific activity 46% higher than the wild type L-ASNase, also presents a reduction of the glutaminase activity by 40% and a decrease of the immunogenic effect of 62.5%, being this a promising enzyme in the pharmaceutical industry (Munhoz Costa et al., 2022). Chemical modifications such as scFv-fusion, TRAIL domain-fusion, albumin binding-fusion, PEGylation, PASylation and bioconjugations have been employed (Guo et al., 2000; Abribat, 2023; Trieu, 2010; Lavie and Nguyen 2017; Brumano et al., 2019). A chemical modification of a commercial biosimilar *E. coli* L-ASNase (Leunase^®^ (Kyowa Hakko Kirin, Japan)) was studied by direct conjugation of carboxyl groups to primary amines by 1-ethyl-3-(3-dimethylaminopropyl) carbodiimide (EDC) (Chahardahcherik et al., 2020). In this case, a polymer called carboxymethyl dextran (CMD) was used, which is biologically compatible. The results showed a substantial increase in the specific activity of the modified L-ASNase compared to the commercial one (1609.62 vs 629.8 U/mg). Additionally, an increase in half-life stability in rat serum of 192 h with the modified L-ASNase *versus* 96 h with the native one, and an improvement in temperature and pH stability were observed. In recent years, bio-better proteins have gained considerable industrial attention, as they are patentable and have higher prices in the market due to their clinical advantages (Brumano et al., 2019).

For large-scale processes, biopharmaceutical production from wild strains host is generally avoided. This is mainly due to low yield and high production costs. To overcome the problems faced by conventional L-ASNase production, one approach would be to use recombinant DNA technology, to transfer genes that encode the enzyme, from one microorganism to another. This is called heterologous expression (Li et al., 2019). Heterologous expression allows the relatively stable, safer expression of enzymes, with higher yields (Patel et al., 2022). There are several expression systems available for biopharmaceutical purposes, including bacteria, yeast, filamentous fungi, mammalian cells, plants, insects, transgenic animals, and even microalgae (dos Santos et al., 2018). Each system has its particular features in terms of production capacity, costs, safety, complexity, and specific processing (Schmidt, 2004). The use of complex and costly expression systems, such as mammalian cells (CHO, insects, etc.), are generally used for proteins that require complex post-translational modifications, which in the case of L-ASNase are not necessary. Concerning the use of plant-based expression systems, these display several disadvantages due to the large numbers of proteases present in their cells, making extraction and purification challenging for large-scale enzyme production (Patel et al., 2022). The high secretors and the host strains of bacteria (e.g., *E. coli, Bacillus* and lactic acid bacteria), filamentous fungi (e.g., *Aspergillus*) and yeasts (e.g., *Pichia pastoris*) are most commonly used for the homologous and heterologous expression of recombinant enzymes without complex post-translational modifications (Goswami et al., 2015). Among these, *E. coli*, *B. subtilis* and *P. pastoris* are used for the production of L-ASNase, since these can quickly and easily overexpress (Wang et al., 2021; Patel et al., 2022). However, the yields of L-ASNase production depend not only on host selection. But also of the fermentation process, and the efficiency of the expression systems (Li et al., 2019). The requirements for a successful high-throughput process for protein production as: 1) high transcription, and translation of genes of specific protein, 2) correct folding, and induction that does not cause stress to the host, 3) desired post-translational modifications, 4) efficient secretion, and limited or no degradation of the product in the culture medium (Kaur et al., 2018).

## 4 Improvement in systems for L-ASNase heterologous expression

As previously mentioned, the design of an efficient bioprocess strategy is essential for a profitable industry of clinically relevant recombinant proteins. Heterologous protein expression using genetically modified prokaryotic hosts has made it possible to provide a wide range of recombinant proteins. This production, would not be feasible without this technology, as the wild-type cells are not prepared to provide it, in a scalable and rentable manner (Kim et al., 2020). Yet, there are challenges and limitations in the use of these systems. Generally, heterologous expression of proteins has various problems, such as: inadequate folding, heavy molecular weight, or the presence of multiple membrane domains in the protein; cellular metabolic burden, codon usage differences, and sequence repetitiveness that affect translation. For example, one study reported that the heterologous expression of l-asparaginase from *Rhizomucor miehei* in *Pichia pastoris* resulted in low protein expression levels and low enzyme activity due to suboptimal transcriptional and translational regulation (Zhang et al., 2021). Effer et al. (Effer et al., 2019) discusses the evaluation of extracellular expression into *P. pastoris* Glycoswitch VR using two different plasmid constructions containing the *asnB* gene (encoding for L-ASNase of *Erwinia chrysanthemi*), with and without His-tag, to find the best system for producing the extracellular and biologically active protein. The study found that the His-tag could negatively affect the tetrameric conformation of L-asparaginase and possibly affect proper protein folding. This could lead to most of the proteins being accumulated for degradation through ER-associated degradation (ERAD), resulting in low extracellular L-asparaginase production. Another study discusses the effect of hydrophobic region on the signal peptide on L-asparaginase secretion and inclusion bodies (IB’s) formation in *E. coli* (Naderi et al., 2022).

**FIGURE 4:**
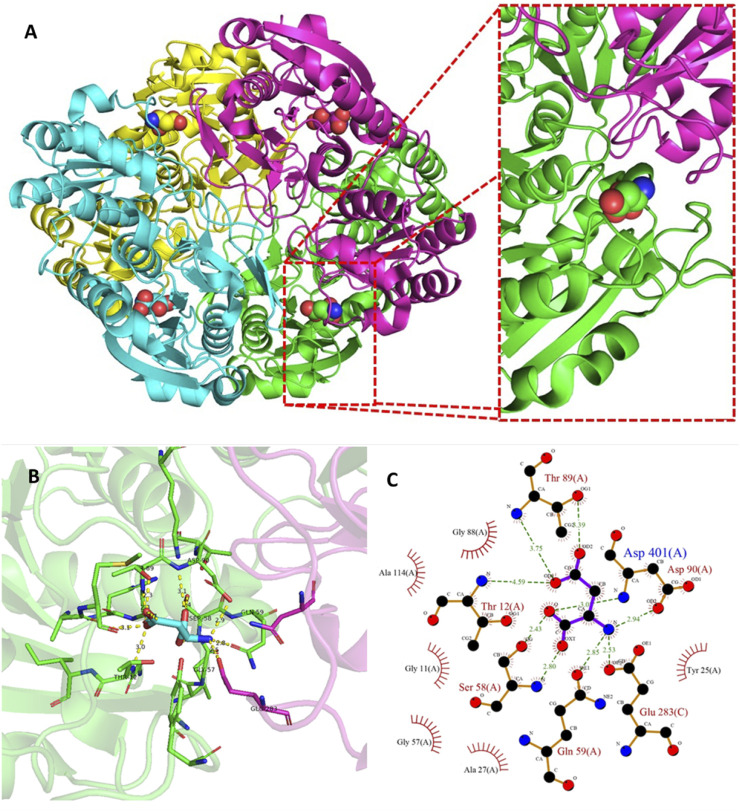
Tetramer structure of L-ASNase and the active site of the enzyme. **(A)** A cartoon representation of the *E. coli* type II L-ASNase homotetramer (PBD 3ECA). **(B)** The active-site pocket which is representative for type I and II L-ASNases. The green monomer represents a major part of the active site, contributing five amino acid side chains directly involved in L-ASNase catalysis. On the other hand, a flexible active site loop is found in the purple monomer, which contains two residues, including the primary nucleophile Thr12. These figures were prepared with PyMOL (Schrödinger). **(C)** Ligand-protein interaction diagram of the l-asparagine binding site generated by LigPlot+ (Laskowski and Swindells, 2011). The interaction of l-asparagine with the 2D residues can be seen. Hydrogen bonds are shown as green dotted lines, while radial arcs represent residues that make non-bonded contacts with the ligand.

Results showed that increasing hydrophobicity of the signal peptides did not necessarily improve secretion efficiency, and in some cases, increased IB’s formation. IB’s are insoluble protein aggregates generated by the metabolic burden that cells undergo upon induction (Vallejo and Rinas, 2004). The problem of IB’s formation or misfolding is further aggravated in the case of L-ASNase. This is mainly because L-ASNase II is fully active in its tetrameric form. This is because the active-site pocket consists mainly of one protomer and is complemented by several residues from the second protomer within a compact dimer (Lubkowski and Wlodawer, 2021) (Figure 4). Therefore, when IB or misfolding occurs, it is required to refold into its native form to maintain its bioactive properties (Mihooliya et al., 2022). Considerable effort has gone into downstream processing involving isolation, solubilization, renaturation (refolding), and purification to obtain the soluble, bioactive protein (Kante et al., 2018). Researchers have achieved up to 50% recovery of functional L-ASNase using various strategies such as the use of strong chaotropic agents (Kante et al., 2018), pulse dilution method (Upadhyay et al., 2014), Freeze-Thaw method (Singhvi et al., 2021), refolding in periodic counter-current chromatography (PCC) (Rajendran et al., 2022), among others. However, these steps are time consuming; require major equipment such as new generation chromatographs, ultrafiltration and diafiltration systems, hydraulic intensifier systems, etc; a large number of reagents such as chaotropic agents, micelles, liposomes, detergents, etc; and use large volumes (generally 1–10 L for mg quantities of protein) (Clark, 2001; Singh et al., 2015; Yuan et al., 2015). Additionally, these strategies are tedious and require large amounts of steps, even more in the case of multimeric proteins, because it requires first a correct renaturation and solubilization of the inactive monomers, for their subsequent refolding of their tetrameric structure under various physiological conditions (Upadhyay et al., 2014; Mihooliya et al., 2022).

**FIGURE 5:**
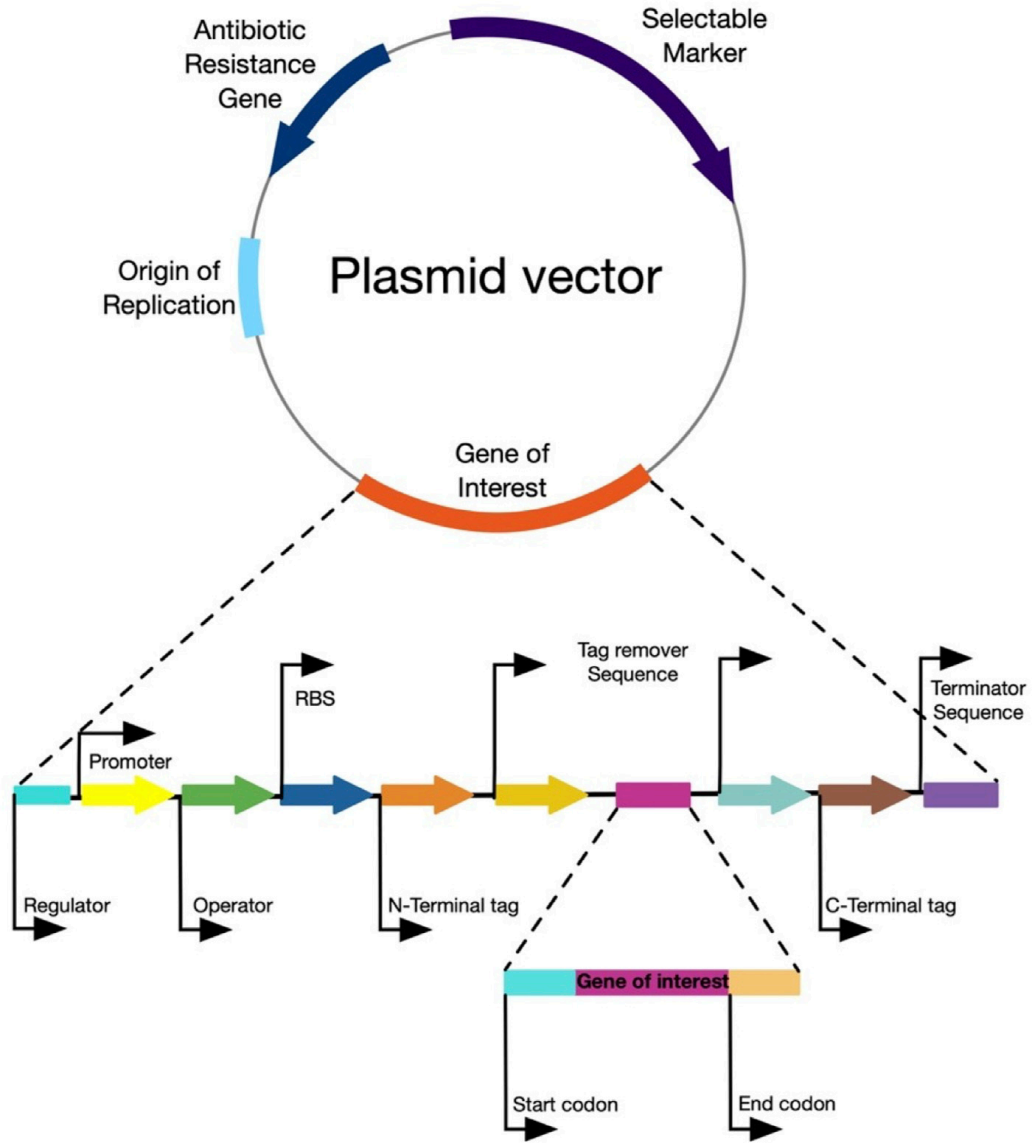
General structure of an expression vector. The figure represents the main components of an ideal expression vector for any host microorganism.

Hence, the rational design of vector systems that include the optimization of codons, transcription regulation, optimization of promoters, translation regulation, the optimization of factors that affect expression in the soluble fraction, co-expression of molecular chaperones and secretion strategies, would bolster the production of the proteins of interest (Juturu and Wu, 2018; Kim et al., 2020; Kant Bhatia et al., 2021) (Figure 5). Table 1 presents the production of heterologous L-ASNase from several sources, together with its expression systems, reviewed in this article.

**TABLE 1:** Production of recombinant L-ASNase and its heterologous expression systems.

Microorganism	Vector	Host cells	Promoter	Secretion signal	Localization		Fermentation type	Enzyme activity	Reference
*Anoxybacillus flavithermus*	pET-22b (+)	*E. coli* BL21-Codon Plus (DE3)-RIL	T7- IPTG inducible	Without signal peptide	Intracellular	NR	SmF	2.5 U/mL	Maqsood et al. (2020)
*Aspergillus terreus*	pET-28a (+)	*E. coli* BL21(DE3)	T7- IPTG inducible	Without signal peptide	Extracellular/periplasmic	Shake-flask	SmF	4.81 U/mg	Saeed et al. (2018)
*Acinetobacter soli* Y-3	pET-30a	*E. coli* BL21(DE3)	T7- IPTG inducible	Without signal peptide	Intracellular	NR	NR	42 U/mg	Jiao et al. (2020)
*Bacillus subtilis* B11-06	pMA5	*B. subtilis* 168	HpaII-constitutive	Without signal peptide	Extracellular/periplasmic	Shake-flask	SmF	9.98 U/mL	Jia et al. (2013)
*Bacillus subtilis* 168	pP43NMK	*B. subtilis* WB600	P43- constitutive	WapA signal peptide	Extracellular	Fed-batch (3L)	SmF	407.6 U/mL	Feng et al. (2017)
*Bacillus licheniformis* Z-1	pP43NMK	*B. subtilis* RIK 1285	P43-constitutive	Native signal peptide	Extracellular/periplasmic	Shake-flask	SmF	426 U/mL	Niu et al. (2021)
*Bacillus licheniformis Z-1*	pP43NMK-BlA-His	*B. subtilis* RIK 1285	PaprE-PyvyD (dual)-constitutivo	Native signal peptide	Extracellular/periplasmic	Batch (4L)	SmF	2163.09 U/mL	Niu et al. (2022)
*Bacillus tequilensis* PV9W	pET-28a (+)	*E. coli* BL21(DE3)	T7- IPTG inducible	Without signal peptide	Intracellular	NR	NR	24.55 U/mL	Shakambari et al. (2018)
*Cobetia amphilecti* AMI6	pQE-80L	*E. coli* BL21(DE3)	T5- IPTG inducible	Without signal peptide	Intracellular	Shake-flask	SmF	778 U/mg	Farahat et al. (2020)
*Erwinia carotovora*	pET30a	*E. coli* C43 (DE3)	T7- IPTG inducible	Without signal peptide	Cytoplasmic	Fed-batch	SmF	0.9 g/L	Roth et al. (2013)
*Erwinia carotovora*	pET-22b	*E. coli* BL21(DE3)	T7- IPTG inducible	PelB	Intracellular	Shake-flask	SmF	16.05 U/mL	Goswami et al. (2019)
*Erwinia chrysanthemi* 3937	Pcrt7/CT-TOPO	*E. coli* BL21 (DE3) pLysS	T7- IPTG inducible	Without signal peptide	Intracellular	NR	NR	25.5 U/mL	Kotzia and Labrou (2007)
*Erwinia chrysanthemi*	pJAG-s1	*Glycoswitch*	AOX1-MeOH inducible	αMF	Extracellular	Shake-flask	SmF	0.456 U/mL	Effer et al. (2019)
		SuperMan5 (his-)							
*Erwinia chrysanthemi*	pJAG_s1	*Glycoswitch*	AOX1-MeOH inducible	αMF	Extracellular/periplasmic	Fed-batch (2L)	SmF	10.7 U/mL	de Almeida Parizotto et al. (2021)
		SuperMan5 (his+)							
*Erwinia Chrysanthemi* NCPPB1125	pPICZαA	*P. pastoris* X33 and *P. pastoris* SMD1168	AOX1-MeOH inducible	αMF	Extracellular	Shake-flask	SmF	1.88 and 3.3 U/mL	Tien Cuong Nguyen (2014)
*Escherichia coli (AnsB)*	pJAG-s1	*Glycoswitch*	AOX1-MeOH inducible	αMF	Extracellular/periplasmic	Shake-flask	SmF	2.98 U/mg	Lima et al. (2020)
		SuperMan5 (his-)							
*Escherichia coli (AnsB)*	pET3a	*E. coli* BL21(DE3)	T7- IPTG inducible	PelB	Periplasmic	Fed-batch	SmF	130 U/mL	Aghaeepoor et al. (2011)
*Escherichia coli (AnsB)*	pET14b	*E. coli* BLR(DE3)	T7- IPTG inducible	PelB	Extracellular	Fed-batch	SmF	870 U/mL	Khushoo et al. (2005)
*Escherichia coli* K12 (*AnsB*)	pET-26b(+)	*E. coli* BL21 star (DE3)	T7- IPTG inducible	PelB+5 aspartate	Extracellular/periplasmic	Shake-flask	SmF	40.8 U/mL	Kim et al. (2015)
Mutant *Escherichia coli*	pET-SUMO	*E. coli* Rosetta	T7- IPTG inducible	Without signal peptide	Intracellular	Fed-batch	SmF	183.5 U/mg	Caetano (2020)
*Escherichia coli* MTCC 739	pPink α-HC	*Pichiapink™*	AOX1-MeOH inducible	αMF	Extracellular	NR	SmF	2.18 U/mL	Sajitha et al. (2015)
*Escherichia* sp. NII	pET-20b	*E. coli* BL21(DE3)	T7- IPTG inducible	PelB	Periplasmic	NR	NR	140 U/mL	Vidya and Pandey (2012)
*Escherichia coli* AS1. 357	pBV220	*E. coli*	P_R_PL-heay inducible	Native signal peptide	Intracellular	NR	NR	228 U/mL	Wang et al. (2001)
*Escherichia* sp	COLADuet-P21285-asn	*E. coli* BL21(DE3)	P21285-IPTG inducible	Without signal peptide	Intracellular	Shake-flask	SmF	3.68 U/mL	Wang et al. (2019)
*Escherichia coli* K-12 (JM109)	pET14b	*E. coli* BL21(DE3)	T7- IPTG inducible	Without signal peptide	Cytoplasmic	Shake-flask	SmF	118 g/L	Upadhyay et al. (2014)
*Escherichia coli* YG 002	pET-15b	*E. coli* BL21(DE3)	T7- IPTG inducible	Native signal peptide	Extracellular	Shake-flask	SmF	17.4 U/mL	Ghoshoon et al. (2015)
*Helicobacter pylori* CCUG 17874	pET-101	*E. coli* BL21(DE3)	T7- IPTG inducible	Without signal peptide	Intracellular	NR	NR	31.2 U/mg	Cappelletti et al. (2008)
*Norcadopsis alba* NIOT-VKMA08	pQE-30	*E. coli* M15	T5- IPTG inducible	NR	Extracellular/intracellular	Shake-flask	SmF	158 U/mL	Meena et al. (2016)
*Pseudomonas fluorescens MTCC 8127*	pET-32a	*E. coli* BL21(DE3)	T7- IPTG inducible	Without signal peptide	Intracellular	NR	SmF	6.4 U/mg	Sindhu and Manonmani (2018)
*Penicillium sizovae*	pPICZαA	*P. pastoris* X33	AOX1-MeOH inducible	αMF	Extracellular/intracellular	Shake-flask	SmF	3 U/mL	Freitas et al. (2022)
*Pectobacterium carotovorum* MTCC 1428	pHT43	*B. subtilis* WB800N	grac-IPTG inducible	amyQ	Extracellular	Shake-flask	SmF	105 U/mL	Chityala et al. (2015)
*Pectobacterium carotovorum* MTCC 1428	pHT43	*B. subtilis* WB800N	grac-IPTG inducible	amyQ	Intracellular	Batch (1L)	SmF	525.98 U/mL	Sushma et al. (2017)
Pyrococcus yayanosii CH1	pMA5	*B. subtilis* 168	P43-constitutive	Without signal peptide	Extracellular/intracellular	Fed-batch (2L)	SmF	5278 U/mL	Li et al. (2019)
*Thermococcus kodakarensis* KOD1	pET-21a	*E. coli* BLR (DE3)	T7- IPTG inducible	Without signal peptide	Intracellular	Shake-flask	SmF	978.7 U/mg (Purified)	Hong et al. (2014)
*Saccharomycescerevisiae* (ASP3)	pPIC9	*P. pastoris* GS115	AOX1-MeOH inducible	α-factor signal peptide	Extracellular/periplasmic	Fed-batch (2L)	SmF	85.6 U/mL y	[ (Ferrara et al., 2006), (Facchinetti de Castro Girão et al., 2016)]
								204.4 U/mg (Purified)	
*Saccharomycescerevisiae* (ASP3)	pPIC9K	*P. pastoris* KM71	AOX1-MeOH inducible	α-factor signal peptide	Periplasmic	Fed-batch (2L)	SmF	3.3 U/mL	Rodrigues et al. (2019)
*Saccharomycescerevisiae* (ASP3)	pPIC9K	*P. pastoris* KM71	AOX1-MeOH inducible	α-factor signal peptide	Periplasmic	Batch (2L)	SmF	0.71 U/mL	Pillaca-Pullo et al. (2021)
*Saccharomycescerevisiae* BY4741 (ASP1)	pET-15b	*E. coli* BL21(DE3)	T7- IPTG inducible	NR	Extracellular/periplasmic	Shake-flask	SmF	196.2 U/mg (Purified)	Costa et al. (2016)
*Yersinia pseudotuberculosis* Q66CJ2	pBAD-24	*E. coli* BL21(DE3)	AraC- arabinose inducible	Without signal peptide	Intracellular	NR	NR	365 U/mL	Pokrovskaya et al. (2012)
*Thermococcus kodakaraensis* KOD1	pET-21b	*E. coli* BL21-CodonPlus(DE3)-RIL	T7- IPTG inducible	Without signal peptide	Intracellular	NR	NR	2350 U/mL (purified)	Chohan and Rashid (2013)
*Rhizomucor miehei* CAU432	pET-28a	*E. coli* BL21(DE3)	T7- IPTG inducible	Without signal peptide	Intracellular	Shake-flask	SmF	1985 U/mg (purified)	Huang et al. (2014)
*Rhizomucor miehei*	pMA5	*B. subtilis* 168	HpaII-constitutive	Without signal peptide	Extracellular	Batch (2L)	SmF	521.9 U/mL	Zhang et al. (2021)
*Thermococcus gammatolerans* EJ3	pET-22b	*E. coli* BL21(DE3)	T7- IPTG inducible	NR	Intracellular	Shake-flask	SmF	7622 U/mg	Zuo et al. (2014)
*Vibrio cholerae*	pMCSG7	*E. coli* BL21(DE3)	T7- IPTG inducible	Without signal peptide	Extracellular/periplasmic	Shake-flask	SmF	821 U/mL	Radha et al. (2018)
*Wolinella succinogenes*	pET28b(+)	*E. coli* BL21(DE3)	T7-IPTG inducible	HB signal peptide	Extracellular/periplasmic	Shake-flask	SmF	238 U/mg	Sannikova et al. (2016)
*Zymomonas mobilis*	pET26B and pET28a	*E. coli* BL21(DE3)	T7-inducible	pelB signal peptide (pET26b)	Extracellular (pET26b) and intracellular (pET28a)	Shake-flask	SmF	0.13 and 3.6 U/mL	Einsfeldt et al. (2016)

SmF: submerged fermentation; NR: not reported.

### 4.1 Codon optimization to improve L-ASNase expression

Messenger RNA (mRNA) of a heterologous gene that contains rare codons can cause significant translation issues, such as stagnation of translation, mRNA and plasmid instability, incorrect amino acid incorporation, displacement of the translation frame, and premature translation completion. In due process, these issues lead to a reduction in the quality and quantity of the synthesized protein (Singha et al., 2017). Codon Optimization can be achieved by replacing rare codons with the original ones, thus adjusting codon bias (Juturu and Wu, 2018).

Codon optimization studies have been performed for the expression of L-ASNase from *Erwinia chrysanthemi* NCPPB1125 in *E. coli* BL21. In this study, codons were optimized and inserted in a pET-21a(+) vector. After adjusting induction and purification conditions, the specific activity reached 312.8 U/mg, which increased 1.5-fold control activity (Nguyen et al., 2016b). Furthermore, the activity of three new uncharacterized extremophilic L-ASNases produced by psychrophilic fungus *Sclerotinia borealis*, thermoacidophilic crenarchaeon *Acidilobus saccharovorans* and thermophilic bacterium *Melioribacter roseu.* The L-ASNase were expressed in *E. coli*, and their codon composition was optimized using the “Twist Codon Optimization” (Twist Bioscience, USA) tool (TwistBioscience, 2022). This strategy allowed to obtain activities of 0.6 U/mL at 24°C and pH 9.6 for *S. borealis*; 2.6 U/mL at 94°C and pH 5.2 for *A. saccharovorans*; and 9.6 U/mL at 37°C and pH 9.6 for *M. roseu* (Dumina et al., 2021).

The L-ASNase of *Z. mobilis* was expressed intracellularly, using pET-28a as vector and extracellularly, using pET-26b, in *E. coli* BL21 (DE3)*.* The yield obtained was 0.13 and 3.6 U/mL, respectively. Results were obtained after codon optimization to better match the host. Therefore, only the extracellularly expressed protein represented an improvement in expression, as native culture in cultures of *Zymomonas mobilis* yield 0.25 U/mL (Einsfeldt et al., 2016)- Recently, a study was conducted *in silico* on the L-ASNase gene of a halophilic bacterium using bioinformatics tools. This study spotted 5 residues associated with rare codons located distant from the active site. These residues can play a role in determining the final structure of the enzyme’s binding site and its substrate (Mortazavi et al., 2020).

### 4.2 Transcription regulation to improve L-ASNase expression

The fine interaction between activator, inducer and repressor molecules, is responsible for the regulation of the biosynthetic pathways at a transcription level. Transcription factors are necessary for the expression and production of enzymes (Kant Bhatia et al., 2021). According to Zhou et al. (Zhou et al., 2019), the transcription strength of a promoter is directly related to its core region (−35 and −10 boxes) for *B. subtilis* hosts, and the optimization of these conserved regions was considered one of the important strategies to increase the yield of recombinant proteins.

A study on L-ASNase production using a dual promoter system, by modifying the −35 and −10 sequences of these promoters, resulted in three mutations. The mutations achieved 6.6%, 7.3%, and 13.3% improvements in expression levels and 4.37-, 4.15-, and 4.86-fold higher transcript intensity compared to the P43 promoter. After 36 h of culture, the expression level in a 10 L fermenter reached 2163.09 U/mL, which was 6.2-fold higher than that of the wild-type strain (based on the P43 promoter) (Niu et al., 2022).

### 4.3 Engineering of promoters to improve transcription of L-ASNase expression systems

The promoter plays a key role in an expression system as its controls the initiation of transcription of the associated genes. An ideal promoter should possess two desirable features: 1) sufficient strength to allow the accumulation of the product up to 50% of total cellular proteins, and 2) strict regulation to prevent product toxicity (Kaur et al., 2018). The choice of promoter depends on the host being used.

In the case of *E. coli*, the main promoters, when expressing a recombinant protein, are derived from bacteria (lac, tac, trp, araBAD) and bacteriophages (T7, T5, SP6) (Kaur et al., 2018). The T7 promoter, derived from bacteriophage T7, is one of the most used promoters due to its extensive use in the pET expression system. Many studies on L-ASNase production are based on the use of pET systems (Khushoo et al., 2005; Kotzia and Labrou, 2007; Cappelletti et al., 2008; Aghaeepoor et al., 2011; Vidya and Pandey, 2012; Chohan and Rashid, 2013; Roth et al., 2013; Hong et al., 2014; Huang et al., 2014; Upadhyay et al., 2014; Zuo et al., 2014; Ghoshoon et al., 2015; Costa et al., 2016; Sannikova et al., 2016; Radha et al., 2018; Saeed et al., 2018; Shakambari et al., 2018; Goswami et al., 2019; Jiao et al., 2020; Maqsood et al., 2020). This is primarily because of the promoter’s efficiency in significantly increasing transcription levels. Several studies have assessed the use of these promoters, and the highest reported L-ASNase reached 978.7 U/mg. This particular L-ASNase is from *T. kodakarensis* KOD1 and was expressed in *E. coli* BLR(DE3) (Hong et al., 2014).

Several studies have been published using promoters other than T7. For example, the production of L-ASNase from *E. coli* AS1.357 in different *E. coli* host strains (JM1105, JM109, TG1, DH5α, and AS1.357) using the pBV220 vector. This vector contains the bacteriophage λ P_R_P_L_ promoter, which is heat-induced. The experiments displayed L-ASNase expression in all the strains examined. However, AS1.357 stood out with the highest expression, achieving an activity of 228 U/mL (Wang et al., 2001). L-ASNase derived from *Cobetia amphilecti* AMI6 was also expressed in *E. coli* BL21 (DE3) using the pQE-80L-kan vector, which features a T5 promoter. They were able to achieve a specific activity of 778 U/mg (Farahat et al., 2020). In another study, using a vector like the previous one (pQE30), L-ASNase from *Nocardiopsis alba* NIOT-VKMA08 was expressed using *E. coli* M15 as the host. In this work, they achieved a high activity of 158.1 U/mL. Nevertheless, developing strategies to synthesize promoters allows for significant upregulation of transcription factors (Meena et al., 2016). A study that following this approach, produced a set of promoters to address the endogenous regulation of different *E. coli* transcription factors (σ ^70^, σ ^38^, σ ^32^, and σ ^24^). Among the designed promoters, P_21285_ was selected as its performance was superior to that achieved with the T7 promoter (Wang et al., 2019).

For the case of *P. pastoris,* promoters for protein expression are limited mainly to the (inducible) AOX1 and (constitutive) GAP promoter. Therefore, for producing L-ASNase using *P. pastoris* as host*,* only pAOX1 is used. The alcohol oxidase І (AOX1) promoter regulates methanol metabolism and initiates the assimilation of methanol, converting it into formaldehyde. Due to its strict regulation and strong inducibility, when methanol is the sole carbon source, it is widely employed to drive heterologous expression (Yang and Zhang, 2018). Numerous studies have been conducted on L-ASNase production using *P. pastoris* as the host system (Ferrara et al., 2006; Tien Cuong Nguyen, 2014; Sajitha et al., 2015; Effer et al., 2019; Rodrigues et al., 2019; Lima et al., 2020; de Almeida Parizotto et al., 2021; Pillaca-Pullo et al., 2021). Among them, a strategy based on methanol-oxygen control in the bioreactor was devised. This strategy produced a 2-fold increase in maximum volumetric activity compared to the pulse strategy (de Almeida Parizotto et al., 2021).

Recently, strategies for L-ASNase production have been developed using *B. subtilis* as host*,* since this microorganism, unlike *E. coli*, is GRAS (generally regarded as safe) due to its non-pathogenic and non-toxic properties. Additionally, being Gram-positive, it allows for the secretion of proteins into the extracellular media (Niu et al., 2021; Souza et al., 2021). Numerous efforts have been made to identify strong promoters for transcriptional control. One of the most extensively studied promoters of *B. subtilis* at an industrial scale for producing L-ASNase is P43, a constitutive promoter considered strong (Feng et al., 2017; Li et al., 2019; Niu et al., 2021). By replacing the HpaII promoter with P43 in *B. subtilis*, improved L-ASNase expression was achieved, resulting in a 38.1% increase in activity. Furthermore, the promoter underwent two rounds of error-prone PCR reactions, leading to random mutagenesis. These variants provided an additional 13% increment in activity compared to P_43_-*B. subtilis* (Feng et al., 2017).

According to Yang et al. (Yang et al., 2021), promoter engineering can modulate the transcriptional capacity of promoters, improving, mutating or changing the DNA sequence of promoters. Using this technique, temperature- and pH-inducible phase-dependent promoters of 114 endogenous promoters were identified and characterized. These were evaluated for the expression of secreted enzymes. This result represents a great potential application for enzyme production, metabolic engineering and synthetic biology (Yang et al., 2017). Using promoter engineering, eight different types of promoters (P_43_, P_yxiE_, P_groEs_, P_sigX_, P_trnQ_, P_131_, P_242_, P_shutttle09_) were evaluated to enhance L-ASNase expression from *P. yayanosii* CH1. A 2.09-fold improvement in transcript levels over the original strain was achieved using the P_43_ promoter and an optimized ribosomal binding site (RBS) (Li et al., 2019). Niu et al. (Niu et al., 2022) developed an approach similar to the one used by Li et al. (Li et al., 2019), but with the difference that they established a dual-promoter system and optimized the core regions (−35 and −10 boxes). The dual-promoter systems performed ideally when nine of the sixteen dual-promoter systems were used (P_aprE_-P_43_, P_yvyD_-P_43_, P_spoVG_-P_43_, P_aprE_-P_aprE_, P_yvyD_-P_aprE_, P_yvyD_-P_yvyD_, P_43_-P_yvyD_, P_aprE_-P_yvyD_, and P_spoVG_-P_yvyD_). This strategy provided greater yields than the original P_43_ promoter. Among these nine systems, the P_aprE_-P_yvyD_ promoter achieved the greatest L-ASNase activity of 502.11 U/mL, which was 1.44 times greater than the activity mediated by the original P_43_ promoter.

### 4.4 Increase in L-ASNase expression by translation regulation

Translation processes are not only responsible for protein synthesis from the mRNA. Also affect folding, structure, and secretion of proteins. To gain greater enzyme production, all mRNA must be translated into proteins and these proteins must be folded into correct structures (Kant Bhatia et al., 2021). To improve the mRNA translation rate and thus increase L-ASNase production, an online RBS calculator is available (DeNovoDNA, 2022), which allows for the design of RBS sequences for *B. subtilis* 168/pMA5-P43-pyasnaseMut. They designed 300 sequences, and theoretically assessed them considering higher yield. Among this sequence, the mutant RBS that achieved the greatest yield was chosen, resulting in a total activity of 5278 U/mL (2-fold higher than the control) (Li et al., 2019). Another free online RBS Calculator, such as “RBS calculator v2.0” (SalisLab, 2022), was used to select a sequence capable of improving expression by 1.39-fold among 22 RBS-assessed sequences (Niu et al., 2022).

Another strategy that improved the production of L-ASNase from *Rhizomucor miehei* was rational design through modification of the 5’ untranslated region (UTR). This region consists of the open reading frame (ORF) and facilitates the accessibility of Shine-Dalgarno sequences and start codons, thus enhancing translation initiation efficiency. By modifying the 5’ UTR, was possible to express a site-directed mutant L-ASNase from *Rhizomucor miehei*, using *B. subtilis* 168 as the host microorganism, resulting in a 6.33-fold increase in L-ASNase activity. The enzyme was produced in high-density batch culture and reached an activity of 521.9 U/mL (Zhang et al., 2021).

## 5 Optimization of factors that affect expression in the soluble fraction of L-ASNase

One of the many approaches to improve the solubility of recombinant proteins, is to slow down the protein synthesis process, thereby allowing sufficient time for the protein to reach its native structure. Some of the strategies for this purpose include using weak promoters, low concentration of inducer and low cell culture temperature (Kant Bhatia et al., 2021). However, all these strategies have the problem of low yield of proteins. Other approaches include the use of genetic engineering to optimize factors such as the co-expression of chaperones and the formation of disulfide bonds, the use of fusion tags, and the translocation of proteins to the extracellular medium (Singha et al., 2017).

### 5.1 Co-expression of chaperones and formation of disulfide bonds in L-ASNase

Co-expression with several types of chaperones involved in protein folding *in vivo* is one of the approaches used to improve the solubility of recombinant proteins (Grigoroudis et al., 2015; Peng et al., 2016; Paraskevopoulou and Falcone, 2018; Wang et al., 2018). Nevertheless, to date only one study has been conducted where they co-expressed chaperones together with L-ASNase (Biglari Goliloo et al., 2021). Biglari Goliloo et al. (Biglari Goliloo et al., 2021) assessed the yield of the co-expression of the GroELS/TF system and L-ASNase (Q59LAsp). Their results showcase that the presence of GroELS and TF chaperones expressed from PG-Tf2 plasmid increased the amount of soluble recombinant Q59LAsp protein in both SHuffle T7 and in *E. coli* BL21 (DE3). In addition, the amount of soluble Q59LAsp protein produced in the SHuffle T7 strain was significantly higher in the presence of chaperones than in *E. coli* BL21 (DE3). This is due to the commercially available SHuffle T7 strain making a chromosome copy of the isomerase with disulfide bond, DsbC along with the *trxB* ^*-y*^ *gor* genotype, which are the genes responsible for providing an oxidative environment, allowing less degradation of L-ASNase (Singha et al., 2017).

### 5.2 Use of fusion tags

In homologous and heterologous expression reactions, it is possible that the final product does not take place in a single step due to the complex coupled reactions; showcasing several limitations in terms of stability, productivity, functional expression and tolerance to intermediaries (Kant Bhatia et al., 2021). Currently, several fusion partner affinity tags are used, which facilitate purification, increase solubility and reduce proteolysis of the recombinant protein (Singha et al., 2017).

Various fusion tags have been reported in the expression of L-ASNase. The maltose binding protein (MBP) of *E. coli*, has been used widely as a fusion partner to increase solubility of recombinant proteins (Dieterich et al., 2003; Kaur et al., 2018). Additionally, the small ubiquitin-like modifier (SUMO) proteins have been used to alter protein properties such as stability and solubility. Caetano (Caetano, 2020), using a pET-SUMO expression system in combination with a mutated L-ASNase sequence from *E. coli,* expressed it achieving an activity of 183.5 U/mg. In 2016, a study was conducted to improve the activity of human L-ASNase hASNase-3, creating a library of mutants using *E. coli* C41 (DE3) as the heterologous expression host (Karamitros and Konrad, 2016a). The expression system used was pET14b-SUMO, improving catalytic efficiency up to 6 times more than the wild enzyme (Karamitros and Konrad, 2014; Karamitros and Konrad, 2016b). Other N-Terminal fusion proteins that have been employed, such as GST (glutathione S-transferase); and affinity tags to facilitate purification (such as Poly-His, which are often used in the L-ASNase expression hosts) (Feng et al., 2017; Effer et al., 2019; de Almeida Parizotto et al., 2021; Niu et al., 2022). Table 2 Summarizes tags commonly used to modify L-ASNase expression systems.

**TABLE 2:** Fusion tag used to improve the solubility of L-ASNase.

Fusion tag	Common expression vector	Description	References
Maltose binding protein (MBP)	pMAL series and pIVEX series	Improves the solubility of the protein	Dieterich et al. (2003)
		Eliminated from the recombinant protein	
		Also aids in purification	
Small ubiquitin-like modifier (SUMO)	pET-SUMO	Promotes folding and structural stability	Caetano (2020)
		SUMO protease enables the elimination of the tag	
Histidine tail (His-tag)	pET	Aids in purification in native or denaturing conditions	[ (Niu et al., 2022), (Freitas et al., 2022), (Chityala et al., 2015)]
	pPICZαA		
	pP43NMK		
	pHT43 series		
Glutathione S-transferase (GST)	pGEX series	Protects against intracellular proteolysis	Darwesh et al. (2022)
		Stabilizes the protein in soluble fraction	
		Also aids in purification	
Thioredoxin (Trx)	pET-32a	Aids in the refolding of proteins that require reducing environment	Sindhu and Manonmani (2018)

In one study, the N-terminal heparin-binding peptide (KRKKKKKGKGLGKKKKR) was used to produce a wild-type L-ASNase derived from Wolinella succinogenes expressed in *E. coli* BL21(DE3). This peptide allows the protein to bind heparin and the cancer cell line K562. The enzyme had two different amino acid substitutions (V23Q and K24) that provide resistance to trypsin lysis. The use of the heparin peptide resulted in an improvement in enzyme activity compared to L-ASNase without the peptide (Sannikova et al., 2016).

### 5.3 Translocation of proteins to the extracellular medium

The efficiency of enzyme production can be limited by their accumulation in inadequate compartments or by inadequate translocation (Kant Bhatia et al., 2021). This limitation can be overcome by slowing down the protein synthesis process, which can also be modified by signal peptides. Secretion facilitates further processing; therefore, in most recombinant production, a secretory signal is cloned along with the gene. This signal can be a native signal or any other efficient signal sequence compatible with the L-ASNase gene frame. In Gram-positive strains like *B. subtilis*, protein secretion is highly efficient and does not require a signal peptide. L-ASNase has been successfully expressed extracellularly in *B. subtilis* through a novel secretion pathway, resulting in a final activity of 426 U/mL. Classical secretion pathways include the Sec-dependent, Tat-translation, and signal recognition particle (SRP) pathways. However, native signal peptides can be replaced with more efficient and validated signal peptides (Niu et al., 2021).

Feng et al. (Feng et al., 2017) succeeded in improving the activity of L-ASNase using *B. subtilis* as the host through combined approaches using combinations of different signal sequences and promoters and using random mutagenesis. In this work, they used eight signal peptides (ywbN, yvgO, amyE, oppA, vpr, lipA and wapA) to assess the amount of protein secretion to the extracellular medium using the HpaII promoter. It was demonstrated that, among the 8 signals, wapA achieved the highest expression, reaching an activity of 407.6 U/mL.

For Gram-negative strains such as *E. coli*, secretion poses a complex challenge. The common scenario is that proteins accumulate in the cytoplasm, which is undesirable for recombinant protein production due to its reducing environment and high concentration of proteases. Moreover, during the extraction process, cell lysis is required, leading to the release of endotoxins and other compounds that complicate purification (Overton, 2014). To enable *E. coli* to express proteins extracellularly, two conditions must be met: 1) maintaining their soluble and active conformation, and 2) providing mechanisms for their delivery into the extracellular space (Kim et al., 2015). Various signal peptides have been employed to facilitate protein translocation to the periplasmic medium, offering a more stable environment. Among these, the signal peptide commonly used in *E. coli* is pelB. For a comprehensive list of articles discussing pelB-based strategies and others, see Table 1. However, new strategies have emerged that further enhance secretion. It has been demonstrated that fusing the pelB sequence with 5 aspartates resulted in nearly double the secretion efficiency of L-ASNase compared to previous approaches (Kim et al., 2015).

Additionally, the secretion of L-ASNase from *E. chrysanthemi* using two signal peptides, OmpA and DsbA, has been investigated (Yari et al., 2020). Signal peptides were selected through an *in silico* approach, taking into account the protein nature, the host organism, and the experimental conditions. Ultimately, it was concluded that DsbA exhibited more efficient targeting of L-ASNase than OmpA (Yari et al., 2020).

The secretory expression of recombinant proteins in yeast necessitates the presence of a signal sequence that facilitates the entry of the recombinant protein into the endoplasmic reticulum (ER), making the initial step for its secretory expression (Yang and Zhang, 2018). The signal sequence of the α-factor of *S. cerevisiae,* along with its truncated versions, has been effectively employed to achieve satisfactory secretion of L-ASNase (see Table 1). In a study, an L-ASNase from *Penicillium sizovae* was expressed using *P. pastoris* as the host organism. The researchers utilized a secretion signal derived from the native α-factor of *S. cerevisiae* to enable efficient secretion of most *P. pastoris* proteins, employing pPICZα as the vector (Freitas et al., 2022).

## 6 Improvement of the host strains to increase the expression of L-ASNase

The choice of host strain can also play a crucial role in the successful protein production process. The selection of strain should primarily consider the requirements of the plasmid expression system, including: 1) the type of polymerases necessary for protein expression, 2) compatibility between available tRNA anticodons and codons of the heterologous gene, 3) stability of the plasmid or protein within the strain, 4) proper protein capability folding within the strain, 5) requirements for posttranslational modifications, and 6) potential toxicity of the protein to the strain itself (Makino et al., 2011). Nowadays, advancements in genetic and metabolic engineering have enabled the modification of organisms to improve their recombinant protein expression levels. The subsequent secretions will discuss the strategies employed in host strain engineering and metabolic engineering that have been utilized to improve the heterologous expression of L-ASNase.

### 6.1 Genetic engineering of host strains to improve the expression of L-ASNase

Targeted strain engineering involves modifying a specific DNA sequence in the host that is known to impact the synthesis, degradation, secretion, or folding of proteins. Several commercial strains of *E. coli, P. pastoris,* and *B. subtilis* have been genetically modified with features designed to improve protein expression. The characteristics and advantages of strains used for L-ASNase production are summarized in Table 3. In the context of L-ASNase expression, 11 *E. coli* expression strains were evaluated. Among them, *E. coli* BL21 ArcticExpress (DE3) demonstrated the best results, producing an enzyme comparable to commercially available ones. This strain presented low protein aggregates, proper folding, and a higher specific activity (156 U/mg) (de Moura et al., 2020). Similar studies have been conducted for the expression of *E. chrysanthemi* in *E. coli*, comparing seven different strains: XL1-Blue, TOP10, UT5600, BL21(DE3), BL21(DE3) Star, Rosetta (DE3), and BL21(DE3) pLysS. Among these strains, *E. coli* Rosetta (DE3) yielded the highest enzyme activity, with 17.8 U/mL in the extracellular medium and 4.2 U/mL in the intracellular medium (Karamitros and Labrou, 2014).

**TABLE 3:** Host expression strains used for the production of L-ASNase.

Host strain	Characteristics	Advantages	Source	References
Host strains of *Escherichia coli*				
*E. coli* BL21(DE3)	Constitutive expression of RNA polymerase T7	Profitable for expression of non-toxic genes	Novagen	Table 1
	Deficient in the Lon and *ompT* genes (proteases)	Stabilizes plasmids		
*E. coli* BL21-CodonPlus (DE3)-RIL	Expresses rare tRNAs; Useful for genes rich in AT content	Allows codon optimization; therefore, expression of the protein	Agilent	[ (Chohan and Rashid, 2013), (Maqsood et al., 2020)]
	Deficient in the Lon and *ompT* genes (proteases)	Profitable for expression of non-toxic genes		
	Constitutive expression of T7 RNA polymerase			
*E. coli* BLR (DE3)	Derived *rec*A from BL21 Constitutive expression of RNA polymerase T7	Stabilizes plasmids that contain repetitive sequences	Novagen	[ (Hong et al., 2014), (Khushoo et al., 2005)]
*E. coli* M15	Constitutively expresses the repressive protein lac	Cannot be infected by lambda phages	Qiagen	Meena et al. (2016)
*E. coli* C41 (DE3)	Mutation in the lacUV5 promoter			
*E. coli* C43(DE3)		Prevents the death associated with toxic proteins	Lucigen	[ (Roth et al., 2013), (Karamitros and Konrad, 2016b), (Karamitros and Konrad, 2014)]
*E. coli* Rosetta	Expression of tRNA for rare codons in *E. coli*	Allows codon optimization; therefore, the expression of the protein	Novagen	Caetano (2020)
*E. coli* BL21 star (DE3)	Mutation in the *rne*131 gene	Improves the stability of mRNA	Invitrogen	Kim et al. (2015)
*E. coli* ArticExpress (DE3)	Expression of genes *cpn*10 and *cpn*60	Improves folding in the cytosol	Agilent	de Moura et al. (2020)
*E. coli* Shuffle T7	Expresses *DsbC* and carries mutations in *trxB* and gor	Promotes correct folding	NEB	Biglari Goliloo et al. (2021)
		Resistant to phage T1		
*E. coli* BL21 (DE3) pLysS	Constitutive expression of T7 lysozymes	Prevents leakage expressions	Novagen	Kotzia and Labrou (2007)
		Improves the expression of genes with toxic inducers		
*Bacillus subtilis* host strains				
*B. subtilis* 168	Wild type	Wild type	ATCC^®^ 23857™	[ (Effer et al., 2020), (Jia et al., 2013), (Li et al., 2019)]
*B. subtilis* WB600	Deficient in *ΔnprE, ΔaprA, Δepr, Δbpr, Δmpr, ΔnprB* (extracellular proteases)	Avoids protein degradation	Wang et al. (2014)	Feng et al. (2017)
*B. subtilis* RIK 1285	Express *trpC2, ys1, aprEdelta3*	Allows high secretion of proteins	Takara	[ (Niu et al., 2021), (Niu et al., 2022)]
	Deficient in *nprR2, nprE18*			
*B. subtilis* WB800N	Deficient in *ΔnprE, ΔaprA, Δepr, Δ bpr, Δmpr, ΔnprB, Δvpr, ΔwprA* (extracellular proteases)	Avoids protein degradation	Murashima et al. (2002)	[ (Chityala et al., 2015), (Sushma et al., 2017)]
*Pichia pastoris* host strains				
*P. pastoris* KM71 (MUT^S^)	Slow growth in methanol	Allows better secretion of complex proteins	Novagen	[ (Pillaca-Pullo et al., 2021), (Rodrigues et al., 2019)]
	Contains a deletion in the histidine gene (arg*4, his4, AOX1::ARG4*)			
*P. pastoris* GS115	Contains a deletion in the histidine gene (*his4*)	Allows better secretion	Invitrogen	[ (Ferrara et al., 2006), (Facchinetti de Castro Girão et al., 2016)]
*P. pastoris* X-33	wild type	Wild type	Invitrogen	[ (Tien Cuong Nguyen, 2014), (Freitas et al., 2022)]
*P. pastoris Pichiapink™*	Contains a deletion in the gene that expresses adenine (*Ade2*)	Allows better secretion	ThermoFisher	Sajitha et al. (2015)
*P. pastoris Glycoswitch*	Contains a deletion in the histidine gene	Allows better secretion	Pichia	[ (Effer et al., 2019), (Lima et al., 2020)]
SuperMan5 (his-)	Interrupts the N-glycosylation pathway of *P. pastoris*, and produces human glycosidic structures. (*his4, och1::pGAPTrα1,2-mannosidase*)	Possible to “humanize” proteins		
*P. pastoris Glycoswitch*	Interrupts the N-glycosylation pathway of *P. pastoris*, and produces human glycosidic structures. (*och1::pGAPTrα1,2-mannosidase*)	Possible to “humanize” proteins	Pichia	de Almeida Parizotto et al. (2021)
SuperMan5 (his+)				
*P. pastoris* SMD1168	Contains a deletion in the histidine gene	Enables the stabilization of proteins	Invitrogen	Tien Cuong Nguyen (2014)
	Does not contain protease A (*his4, pep4*) activity	Allows better secretion		

### 6.2 Metabolic engineering in hosts to improve the heterologous expression of L-ASNase

The overexpression of recombinant proteins triggers a cellular stress response (CSR). This response is primarily caused by the diversion of energy and metabolites, including amino acids, ribosomes, and other precursors, towards protein synthesis (Munhoz Costa et al., 2022). Therefore, gaining a better understanding of CSR and developing strategies to control it are crucial for successful recombinant protein production.

Global regulators within the CSR transcriptional regulatory network were discovered by L-ASNase expression in *E. coli*. Specifically, the group of regulators having the greatest impact on gene expression in the regulatory network was identified and their influence on synthesis was assessed. By biological and bioinformatic analyses, it was determined that genes suppressed by *fis*, such as *carB*, *fadB, nrfA, narH* and *queA,* are also activated during the stationary phase. Consequently, this might be considered a possible target for modulating metabolic activity and capacity for protein expression. When *fis* was co-expressed together with L-ASNase at 6 h of induction, the volumetric efficiency of L-ASNase increased 3-fold, compared to the native form of the host (Mahalik et al., 2017). The role of the *lrp* gene was also evaluated, concluding that its co-expression is a suitable target to enhance expression. Achieving a maximum volumetric efficiency of 458.43 mg/L, this result in a 1.5-fold improvement compared to the native expression level (Mahalik et al., 2022). In another study conducted by Sharma et al. (Sharma et al., 2020), studied post-induction upregulated genes as potential candidates for the generation of Cellular Stress Response (CSR) using *E. coli* as strain model. To do this, they evaluated four main double knockouts (∆elaA + ∆cysW, ∆elaA + ∆cueR, ∆cysW + ∆purL and ∆yabI + ∆cysW) and six main single knockouts (control strain, ∆purL, ∆elaA, ∆cysW, ∆cueR, ∆cysJ and ∆yfbN), where they transformed pMAL-p2X plasmid with the L-ASNase gene (*ansB*) cloned under the tac promoter. Double mutants yielded better results, the best of which ∆elaA + ∆cysW improved the activity 2.32-fold over the control strain. Thus, the knock-out strategy would allow the creation of more efficient hosts for L-ASNase production.

## 7 Future challenges for the rational design of heterologous systems for L-ASNase expression

To achieve successful L-ASNase production, it is crucial to employ a rational approach in designing expression systems, selecting appropriate strains, and making genetic or metabolic modifications. While various strategies have been explored, such as strain engineering, metabolic engineering, and bioinformatics tools, there is still room for improvement in L-ASNase production. Cutting-edge computational, such as In Silico Optimization (ISO) tools, are being adopted to enhance the process. These tools utilize computational methods like simple algorithms, dynamic programming, statistical techniques, and machine learning algorithms (such as artificial neural networks, support vector machines, and deep learning) to generate comprehensive models, reducing the need for time-consuming *in vitro* experimentation (Packiam et al., 2020), *In Silico* Optimization (ISO) tools use appropriate computational methods to generate models based on these approaches, which allow tackling the optimization with a broader and integrative view. Thus, avoiding *in vitro* experimentation and in the process, speeding up the workflow. Examples of computational methods include: 1) simple algorithms, 2) dynamic programming, 3) statistical methods, and iv) ISO tools offer several benefits for optimizing gene expression, allowing modifications based on the host. They evaluate and adjust gene properties like codon usage, GC composition, mRNA stability, cryptic splice sites, and premature polyadenylation signals (Watts et al., 2021; Vasina et al., 2022). Notable tools in this area include “SignalP” and “Phobius,” which predict the most efficient signal peptide for a given amino acid sequence, thereby saving time and resources by eliminating the need for constructing multiple vectors (Zhou et al., 2018). Another approach involves using the “nondominated sorting differential search algorithm and flux balance analysis (ndsDSAFBA)", a multi-object optimization model that leverages *in silico* metabolic pathway models to enhance metabolite production. This approach offers a less labor-intensive and cost-effective methodology (Daud et al., 2019) Additionally, deep neural networks have been applied, such as the “mutation predictor for enhanced protein expression (MPEPE)", which can suggest amino acid sequence mutations to improve protein expression (Ding et al., 2022). Moreover, machine learning (ML) is being employed as a rational design strategy, exemplified by the development of MALLPHAS, a strain engineering tool that optimizes protein secretion (Markova et al., 2022).

Another recent strategy involves the use of CRISPR-Cas-based gene editing tools to enhance recombinant protein expression (Gu et al., 2018; Baghban et al., 2019; Fontana et al., 2020). CRISPR has been employed as a synthetic promoter activator for optimizing protein expression. Additionally, small molecule-sensitive gRNAs have been utilized to regulate gene expression in *E. coli* and precisely control multigene pathways (Fontana et al., 2020). The application of CRISPR-Cas9 for genomic engineering in yeast, including *P. pastoris*, has been reported, enabling rapid and marker-free modifications for strain and metabolic engineering purposes (Weninger et al., 2016). *B. subtilis* is another microorganism benefiting from CRISPR technology, with the development of a CRISPR-Cas9 toolkit for comprehensive engineering. This toolkit addresses challenges like low editing efficiency, complex cloning processes, and limited multiplexing capacity, thereby advancing the engineering capabilities of this strain (Gu et al., 2018). The combination of CRISPR and machine learning (ML) facilitates the maturation of metabolic engineering. CRISPR technology enables modifications at numerous genomic sites, simplifying gene editing and metabolic perturbations. ML, on the other hand, aids in the rational selection of optimal genes for desired products or applications through predictions and recommendations (Lawson et al., 2021).

The future challenges in optimizing heterologous protein expression involve integrating the aforementioned bioinformatics tools and designing tools capable of optimizing expression systems comprehensively, from transcriptional regulators to transcription termination. Furthermore, emerging technologies like CRISPR and ML hold promise in facilitating optimization and enhancing the reliability of predictions. In the coming years, the synergy between new algorithms and biotechnological tools should enable the development of advanced software and methodologies that can significantly reduce the time and costs associated with pharmaceutical products like L-ASNASA.

## 8 Conclusion

Overall, this review provides an overview of molecular and metabolic strategies that can be used to optimize heterologous expression of L-ASNase. This article describes several approaches that have been employed for this means, including the use of molecular tools, strain, and metabolic engineering, and *in silico* optimization. Through a clear and insightful analysis, it highlights the need for a rational design approach to achieve successful expression. In addition, it acknowledges the challenges to large-scale production of bio-betters L-ASNases.

**FIGURE 6:**
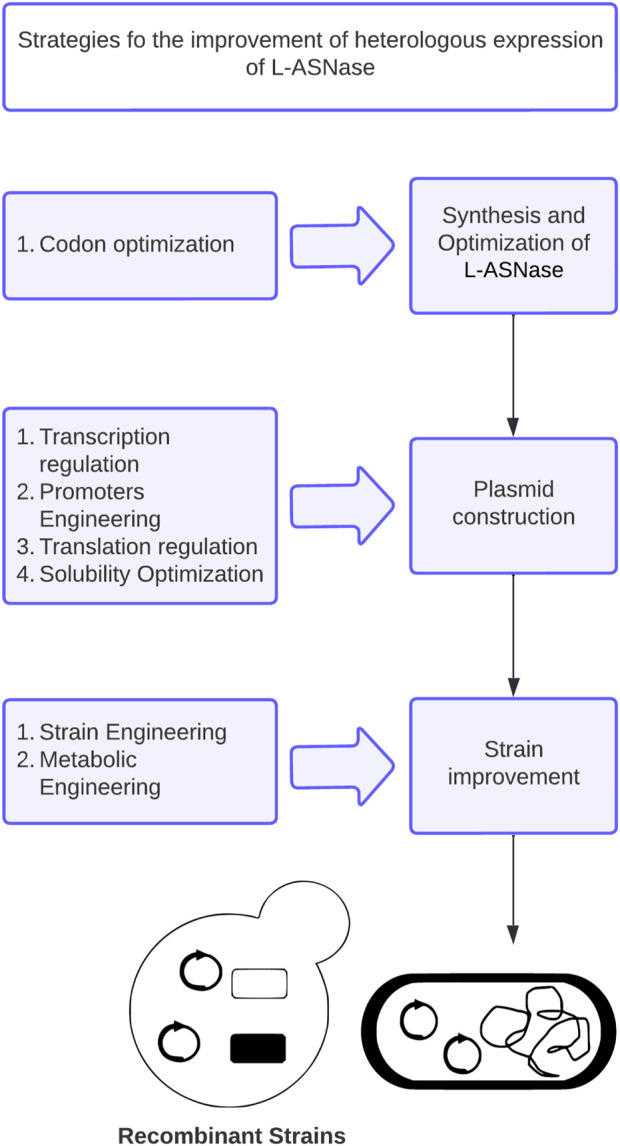
Proposed workflow to improve heterologous expression of L-asparaginase.

In summary, the use of genetic engineering, rational design of heterologous expression systems, metabolic strategies, would allow motivating and facilitating the pharmaceutical industry to continuously innovate product manufacturing processes, and develop new treatments effectively and using Good Manufacturing Practices (GMP). In this sense Brumano et al. (Brumano et al., 2019), mentions that the development of bio-betters L-ASNases begins with the development of the process. Therefore, the search for hosts and expression systems that facilitate upstream and downstream processes such as 1) expression systems and the use of bioinformatics tools that allow codon optimization such as “Twist codon optimization” and RBS such as “RBS calculator v2. 0”; 2) hosts capable of producing l-ASNases from new microbiological or molecularly and/or chemically modified sources; 3) enzyme secreting microorganisms such as *B. subtilis* or *P. pastoris* strains; 4) expression systems capable of stabilizing structure conformation and solubility such as the use of pET-SUMO systems or co-expression of chaperones; and 5) that manage to improve L-ASNase production yields such as promoter optimization have been reviewed in this work. These strategies would guarantee the efficacy and safety of the final product that L-ASNase producing industries seek for a continuous improvement of both the process and the product. For this reason, it is of utmost importance to develop a strategy to address all the points mentioned in this review. In Figure 6 it proposes a workflow that would allow an effective rational design of the host and expression system to produce biologically improved L-ASNase.
